# Non‐linear shrinkage of Batson's #17 resin during vascular corrosion casting

**DOI:** 10.1111/joa.13718

**Published:** 2022-06-25

**Authors:** Kang‐Chi Julia Shih, Véronique Peiffer, Ethan M. Rowland, Peter Sowinski, Peter D. Weinberg

**Affiliations:** ^1^ Department of Bioengineering Imperial College London, South Kensington Campus London UK; ^2^ Department of Aeronautics Imperial College London, South Kensington Campus London UK

**Keywords:** arteries, Batson's #17, corrosion casting, resin, shrinkage

## Abstract

Many studies of cardiovascular function require a realistic representation of vascular geometry. Corrosion casting has been used to acquire such geometries for many decades. However, the fidelity with which this method reproduces vascular anatomy has not been completely determined. Here we report on the non‐linear shrinkage characteristics and exothermic properties of Batson's #17, a widely used casting resin, in model systems and in aortas of rats and rabbits. The setting process was captured using high‐resolution photography. Shrinkage ranged from 3.4 ± 1.5% of the diameter in 1 ml plastic syringes (inner diameter 4.8 mm) to 19.6 ± 5.6% in the aorta of rats (diameter 1.5–2.6 mm). In addition, aortic curvature and branching angles changed during setting. These effects should be determined and corrected in studies of vascular geometry where high accuracy is required.

## Graphical abstract

Vascular corrosion casting is frequently used to characterize arterial geometry. Here, we quantify the non‐linear shrinkage of the casting resin that can occur during such procedures.

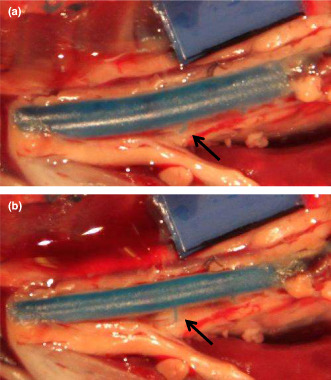

## 1 INTRODUCTION

Reconstruction of vascular geometry is an important step in many studies related to the cardiovascular system. For example, an accurate representation of the lumen is crucial in the computation of arterial blood flow (Steinman, 2004). In situ vascular corrosion casting is the preferred method among *post‐mortem* techniques (Kratky et al., 1989, Moore et al., 2009). Casting materials can be based on silicone rubbers (e.g. Microfil; Flow Tech Inc., Carver, MA, USA) (Hoi et al., 2011), epoxies (e.g. araldite) (Hanstede & Gerrits, 1982), or methacrylate (e.g. Batson's #17 resin; Polysciences Inc., Warrington, PA, USA) (Vincent et al., 2011, Peiffer et al., 2012). We focus on the last of these, which was introduced over 65 years ago (Batson, 1955) and has been in widespread use ever since.

Methacrylates increase in density during polymerization and hence shrinkage occurs (Schricker, 2017). Kratky and Roach (1984) showed that a mixture based on Batson's #17 gave a 20 ± 0.7% volume shrinkage in femoral arteries of sheep compared to 15.8 ± 0.4% in rigid syringes, whilst for different methyl methacrylate‐based resins, Weiger et al. (1986) found the combined shrinkage of all but the largest vessels of the rat to be 8.0 ± 0.8% by volume. The aim of the present study was to increase understanding of the shrinkage behavior of Batson's #17 resin. Non‐linearities and temperature effects were investigated in vitro in rigid syringes and flexible tubing of various sizes, and in situ in the rat and rabbit aorta.

## 2 MATERIAL AND METHODS

### 2.1 General experimental setup

Batson's #17 resin was prepared using a 60:13:3 ratio by weight of monomer base solution, catalyst, and promoter, respectively. Batson's #17 blue pigment, was added according to the manufacturer's instructions.

Shrinkage characteristics were determined by high‐resolution photography (Canon EOS 500D SLR or Sony MVX35i cameras, both with f:3.5–5.6 zoom lens; 3200 × 4700 or 4600 × 3000 pixels, respectively). The number of pixels was converted to an absolute length using the dimensions of an object of known size in the image.

Images were taken at least immediately after the resin was injected, and after the resin had completely solidified. For a number of samples, the complete shrinkage process was tracked by taking consecutive images every minute for approximately 1 h. Images were processed using ImageJ. The percentage shrinkage in diameter was calculated as follows:

$$\frac{D_{\text{final}}-D_{\text{initial}}}{D_{\text{initial}}}*100$$

where *D*_initial_ and *D*_final_ are the cast diameters before and after resin setting, respectively. An analogous formula was used for lengthwise shrinkage. Measurements of the set casts were verified using vernier calipers.

### 2.2 Casting in rigid syringes in vitro

Liquid resin was drawn into polypropylene syringes (1–50 ml; Becton Dickinson). The syringe tip was then sealed and the plunger removed. The syringe was placed vertically (tip down) in a stand. In 7 experiments, a thermocouple temperature probe (Lascar Electronics) was inserted in the resin mixture immediately after the resin was drawn into the syringe. Temperature was measured every 30 s for approximately 70 min, by which time the resin had set. Ambient temperature was 21–22°C.

### 2.3 Casting in flexible tubing in vitro

Eight experiments were conducted with polyvinyl chloride tubing (internal diameter 3.00–6.35 mm) placed in a horizontal position. Liquid resin was injected into the tube via a three‐way tap, which was also connected to a manometer. Once a segment of ~20 cm was filled, the tubing was clamped and the pressure of the closed system was brought to 50 or 100 mmHg. Pressure was maintained until the resin had set completely.

### 2.4 Casting in arteries in situ

All animal procedures complied with the Animals (Scientific Procedures) Act 1986 and were approved by the local ethical review panel of Imperial College London under Home Office licence PPL 70/7333. Nine rats and three rabbits were used. Rats were euthanized by CO_2_ inhalation, and rabbits with sodium pentobarbitone (Euthatal, 160 mg/kg, IV) after receiving heparin (2000 USP units, IV).

Each animal was placed in the supine position and its thorax and abdomen were opened along the ventral midline. The descending aorta was cleared of adherent connective tissue. A retrograde cannula was then tied into the abdominal aorta. The vascular system was flushed with saline after puncturing the right ventricle to act as a drain for the perfusate. Resin was injected at a pressure of 100 mmHg. The infusion was continued, maintaining the pressure, until the resin started to solidify and no more resin could be added. Throughout, the aorta was imaged by a camera facing downwards. Shrinkage was computed for two to three points along the vessel (inter‐point spacing of ~6 mm).

### 2.5 Statistics

Data are presented as mean ± SD. Differences were analyzed by Student's unpaired t‐test, using *p* < 0.05 as the criterion of significance. Coefficients of determination (*r* ^2^) were calculated where appropriate.

## 3 RESULTS

### 3.1 Casting in syringes

**FIGURE 1:**
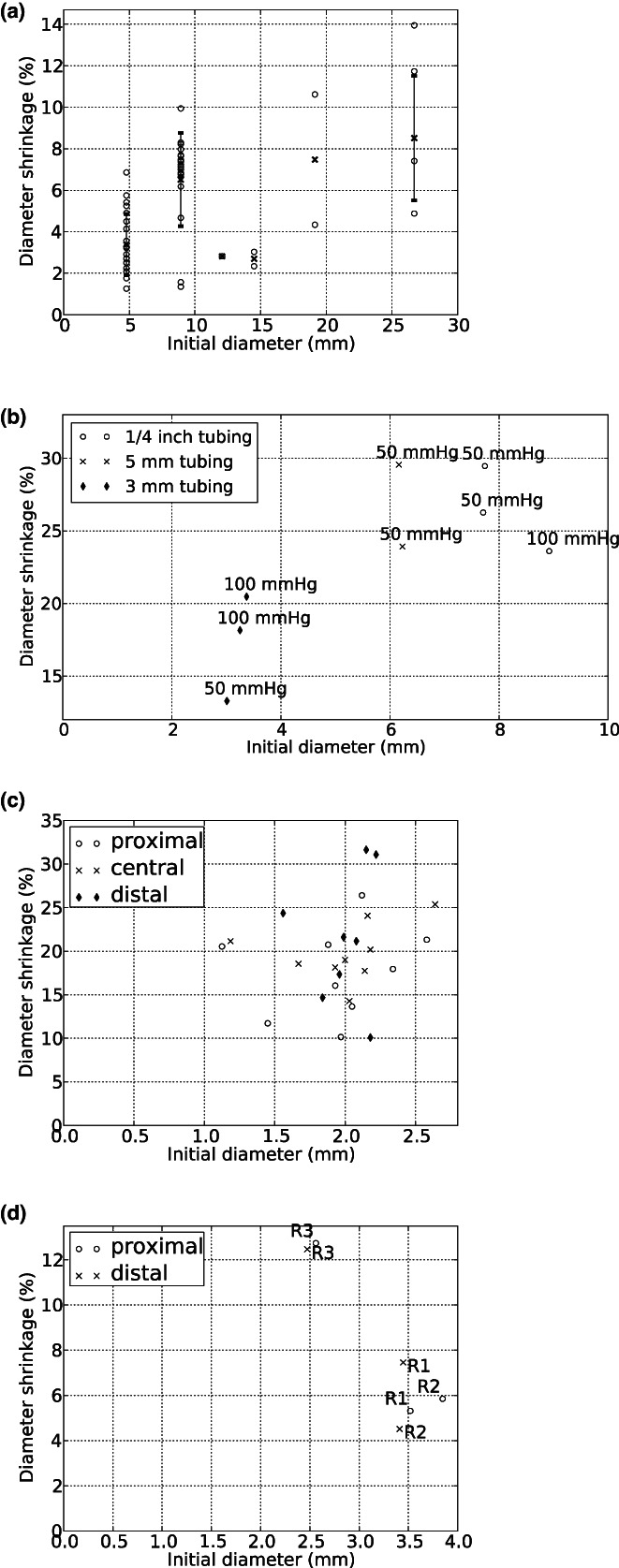
Percentage shrinkage in diameter measured during casting in rigid syringes (a), flexible tubing (b), rat aortas (c) and rabbit aortas (d). In (a), open circles represent individual measurements, and vertical lines show mean (indicated with a cross) ± 1 SD.

When resin was left to set in a rigid syringe with the plunger removed, it shrunk both in diameter and in length. The resulting casts were circular in cross‐section. For 1 ml syringes, which are closest in size to the largest arteries of rats and rabbits, shrinkage was 3.4 ± 1.5% in diameter and 14.6 ± 3.1% in length. Figure 1(a) summarizes the amount of diameter shrinkage in syringes of various sizes. Diameter shrinkage in 1 ml syringes was significantly different from the diameter shrinkage in 2 ml syringes (6.5 ± 2.3%, *p* = 3 x 10^−6^), indicating non‐linear behavior. The trend for increased percentage shrinkage with increased initial diameter appears to continue in Figure 1(a).

**FIGURE 2:**
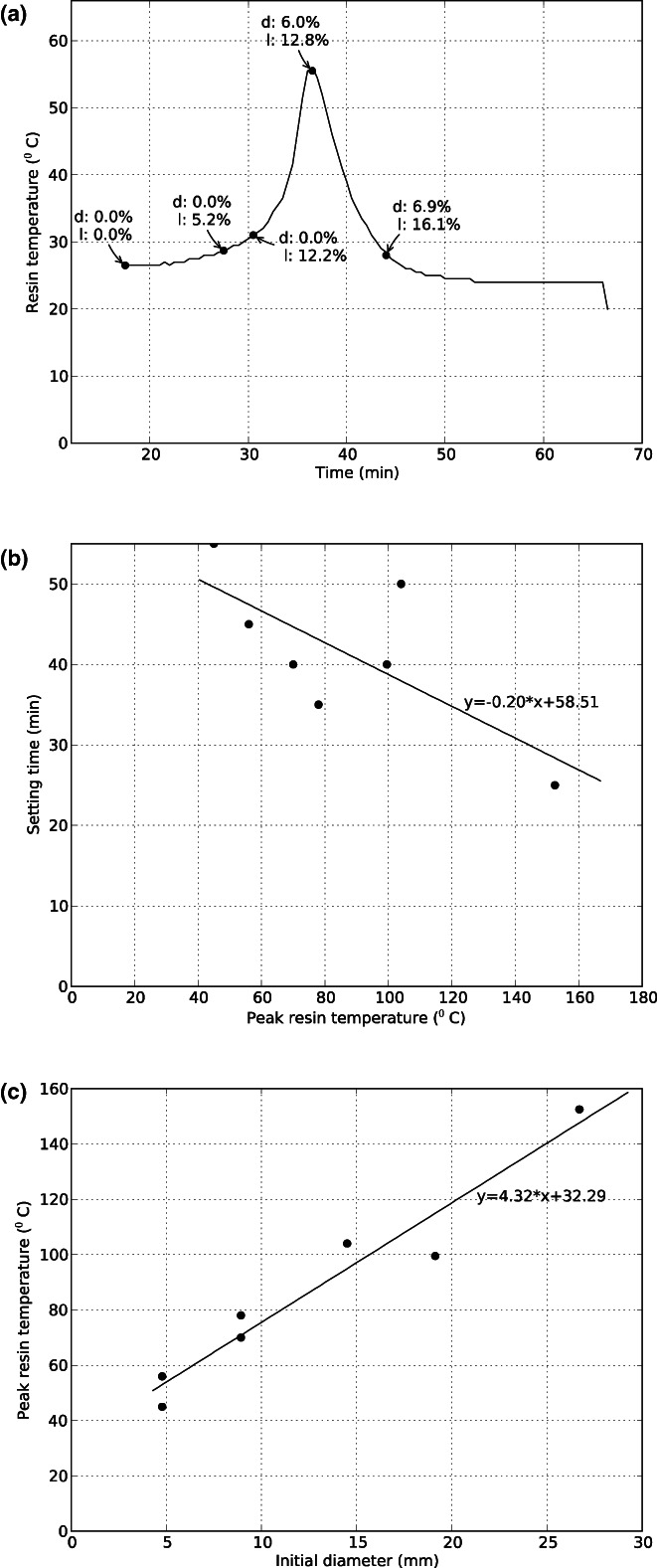
(a) Resin temperature as a function of time during casting in a 1 ml syringe. Resin components were mixed at *t* = 0. The amount of shrinkage in diameter (d) and length (l) are indicated for various points during the process. (b) Setting time versus peak resin temperature during casting in syringes (*r* ^2^ = 0.52, *p* = 0.067); (c) Peak resin temperature during casting in syringes showed a strong linear correlation with syringe diameter (*r* ^2^ = 0.94, *p* = 0.006).

The evolution of resin temperature and shrinkage with time is shown in Figure 2(a) for an experiment in a 1 ml syringe. The majority of the shrinkage in diameter occurred around the peak in temperature; shrinkage in length commenced earlier. Faster setting was associated with higher temperatures, and peak resin temperature depended linearly on syringe diameter (*r* ^2^ = 0.94, *p* = 0.006) (Figure 2(b,c)).

### 3.2 Casting in flexible tubing

Casts made in flexible tubing were approximately oval in cross section. Shrinkage was therefore assessed by averaging 2 orthogonal diameter measurements made with calipers. Figure 1(b) summarizes diameter shrinkage for experiments with tubing under different pressures. There was an upwards trend in the plot of shrinkage vs initial diameter, and shrinkage was markedly higher than in syringes of similar diameter. The results did not suggest any dependence of shrinkage on infusion pressure.

### 3.3 Casting in arteries

**FIGURE 3:**
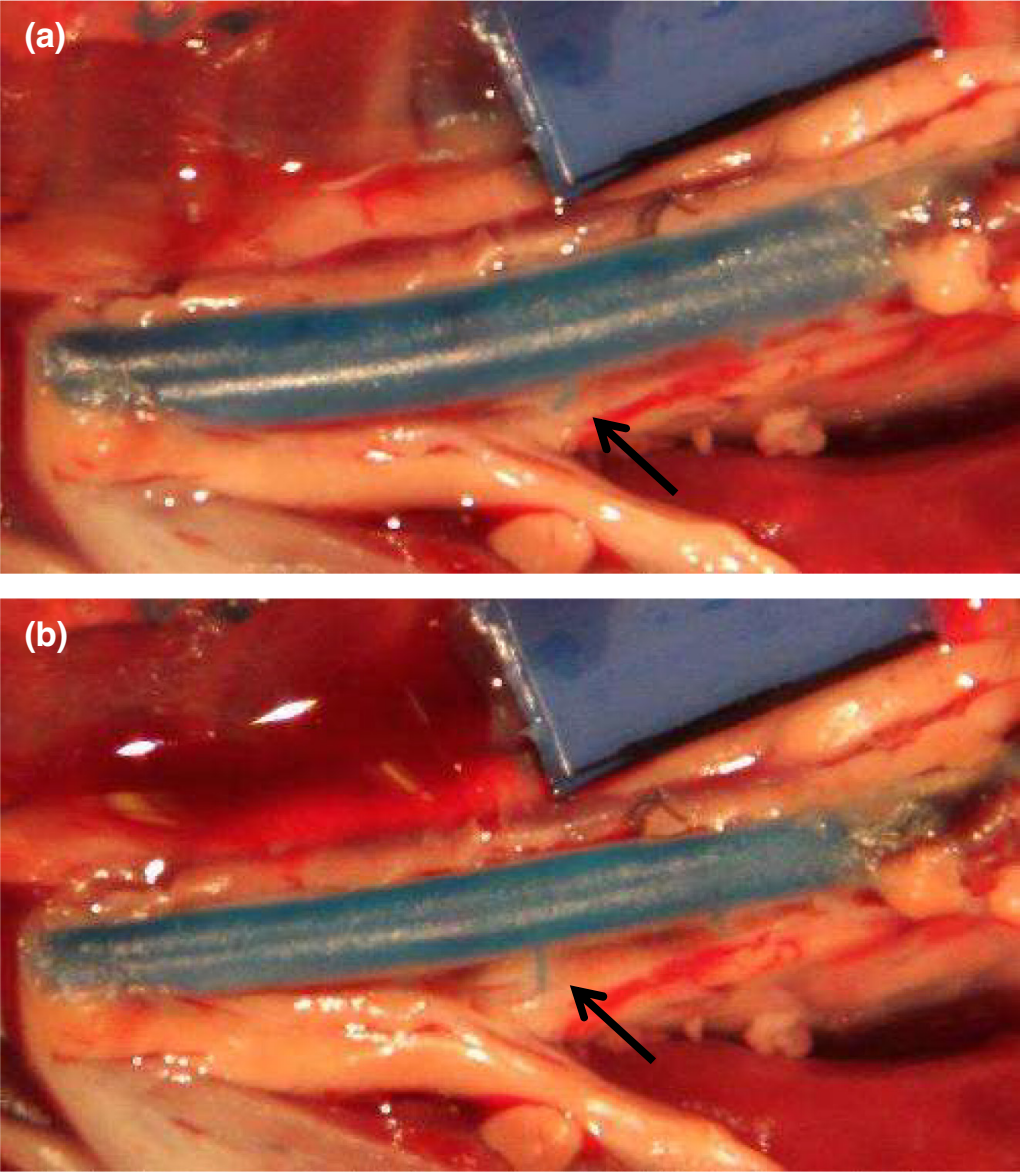
Images taken before (a) and after (b) resin setting in a rat aorta, showing how the vessel straightened during the setting process. The arrows point to an intercostal artery, whose branching angle increased over time.

Rat aortic diameters ranged between 1.6 and 2.5 mm. Casts shrunk by 20.6 ± 3.3% in diameter. There was a tendency for greater shrinkage in bigger vessels but it did not reach statistical significance (*p* = 0.12, Figure 1(C)). Figure 3 illustrates how the aorta straightened and how branching angles increased during the setting process in one animal. Diameters of the rabbit aortas, averaged over 2 measurement points, ranged between 2.5 and 3.6 mm. Shrinkage for this species was 8.1 ± 4.0% in diameter (Figure 1(d)), which is less than in rat aortas despite the larger diameter.

## 4 DISCUSSION

Shrinkage in 1 ml syringes (3.4 ± 1.5% in diameter and 14.6 ± 3.1% in length) was within the range reported by Kratky and Roach (1984) [13] for the same container but a different composition of Batson's #17‐based resin. We additionally found that shrinkage in syringes was non‐linear, with a significantly greater percentage shrinkage in 2 ml syringes than in 1 ml syringes.

Diameter‐wise shrinkage in flexible tubing was proportionally greater than in similarly sized rigid syringes, and different again – and species dependent – in aortas: 8.1 ± 4.0% in the rabbit, and as much as 20.5 ± 3.3% in the rat aorta. Differences could in part be due to the difference in set‐up: syringe experiments used open systems, experiments in tubing used closed systems but flexible tubing, and the animal experiments had leakage from the region of interest but, as with the flexible tubing, pressure was maintained until the resin was sufficiently set that no more would enter.

Caliper measurements of the casts made in flexible tubing revealed that cross‐sections were approximately oval. This was probably caused by a gravitational effect: the tubing was placed horizontally whilst the resin set. Similar phenomena may occur when casting arteries. Further distortions were observed in the in situ experiments: curved vessel segments tended to straighten and branching angles changed during setting. This observation might also explain discrepancies between the iliac bifurcation angles measured by MRI and by vascular casting (Moore et al., 1999).

In conclusion, we suggest the use of methacrylate formulations that reduce shrinkage (e.g. Schricker, 2017), and/or the use of higher infusion pressures to compensate for shrinkage. Ideally, the pressure should be determined from pilot experiments in which dimensions before and after resin setting are monitored in the animal model and vascular section of interest.

## AUTHOR CONTRIBUTIONS

Kang‐Chi Julia Shih, Ethan Min Rowland, and Peter Sowinski conducted the experiments and Kang‐Chi Julia Shih, Veronique Peiffer, and Peter D. Weinberg wrote the manuscript.

## FUNDING INFORMATION

This work was funded by the British Heart Foundation and the British Heart Foundation Centre of Research Excellence.

## Data Availability

The data that support the findings of this study are available from the corresponding author upon reasonable request.
